# Antibiofilm properties of chemically synthesized silver nanoparticles found against *Pseudomonas aeruginosa*

**DOI:** 10.1186/1477-3155-12-2

**Published:** 2014-01-14

**Authors:** Navindra Kumari Palanisamy, Nas Ferina, Athirah Nur Amirulhusni, Zaini Mohd-Zain, Jamal Hussaini, Liew Jian Ping, Rajkumar Durairaj

**Affiliations:** 1 Faculty of Medicine, Universiti Teknologi MARA (UiTM), Jalan Hospital, Sg. Buloh, Selangor 47000, Malaysia; 2 Faculty of Engineering and Science, University Tunku Abdul Rahman Jalan Genting Kelang, Kuala Lumpur, Setapak 53300, Malaysia

**Keywords:** Antibacterial activity, Silver nanoparticles, Biofilm, Inhibitory effect, Multidrug resistant *Pseudomonas aeruginosa*

## Abstract

Nanomedicine is now being introduced as a recent trend in the field of medicine. It has been documented that metal nanoparticles have antimicrobial effects for bacteria, fungi and viruses. Recent advances in technology has revived the use of silver nanoparticles in the medical field; treatment, diagnosis, monitoring and control of disease. It has been used since ancient times for treating wide range of illnesses. Bacterial cells adheres to surfaces and develop structures known as biofilms. These structures are natural survival strategy of the bacteria to invade the host. They are more tolerant to commonly used antimicrobial agents, thus being more difficult to be controlled. This leads to increase in severity of infection. In this study, we have investigated the effect of silver nanoparticles in the formation of biofilm in multidrug resistant strains of *Pseudomonas aeruginosa*. Observation showed that biofilm formation occurred at bacterial concentration of 10^6^ cfu/ml for the sensitive strain of *P. aeruginosa* while in the resistant strain, the biofilm was evident at bacterial concentration of about 10^3^ cfu/ml. The biofilm were then tested against various concentrations of silver nanoparticles to determine the inhibitory effect of the silver nanoparticles. In the sensitive strain, 20 μg/ml of silver nanoparticles inhibited the growth optimally at bacterial concentration of 10^4^ cfu/ml with an inhibition rate of 67%. Similarly, silver nanoparticles inhibited the formation of biofilm in the resistant strain at an optimal bacterial concentration of 10^5^ cfu/ml with an inhibition rate of 56%. Thus, silver nanoparticles could be used as a potential alternative therapy to reduce severity of disease due to *P. aeruginosa* infections.

## Background

*Pseudomonas aeruginosa*, a gram negative bacterium, is an important opportunistic pathogen. It has been reported to be resistant to commonly used empirical antibiotic treatment and has been documented to be responsible to high rates of morbidity and mortality [1]. *P.aeruginosa* is the causative pathogen for several infections which includes urinary tract infection, septicaemia, osteomyelitis and endocarditis. Thus, it poses new challenges as the emergence of multidrug resistance strains is at alarming rates.

Nanoparticles are defined as nanoscale particles with novel and distinctive physicochemical properties [2]. Today, nanotechnology has been used in many different applications such as in the medical field; imaging and medical apparatus [3], sensors [4], fabrics, cosmetics, health products, and water remediation technologies. Silver based topical dressing has been widely used as a treatment for infections in burns, open wounds and chronic ulcers [5]. Recently, silver nanoparticles has been used as a molecular tool and method for targeted drug delivery [6]. Currently, nanoantibiotics is seeking our attention as it could be used as an alternative therapy. Numerous literatures has reported the use as silver nanoparticles as an antimicrobial agent. This includes the report by Kim Kuk et al. [7] who documented the antimicrobial effect of silver nanoparticles in yeast, *Escherichia coli* and S*taphylococcus aureus*[7]. There are also other researchers who investigated the similar effect in *Bacillus megaterium*, *Staphylococcus aureus, Escherichia coli, Proteus vulgaris* and *Shigella sonnei*[8,9]. However, very minimal work has been carried out in multidrug resistant strains of *P. aeruginosa*.

**Figure 1:**
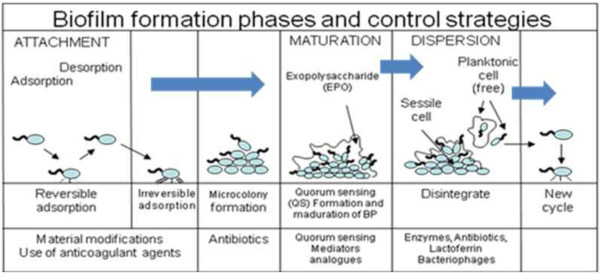
**Development of biofilm [**[15]**].**

Biofilms are bacterial communities that colonize and are found embedded, in a self-produced exopolysaccharide matrix. They attach to surfaces or living tissues [10]. Formation of biofilms allows organisms to survive and thrive in hostile environment, disperse to form new niches and gives them significant advantages in protection against environmental fluctuations such as humidity, temperature, pH, the concentration of nutrients and waste removal [11,12]. Biofilms has been a problem in the medical field, especially in delaying the process of wound healing. The presence of biofilm has been associated with various diseases, i.e.: endocarditis, otitis media, chronic prostatitis, cystic fibrosis, periodontitis, infections caused by medical devices and nosocomial infections [13,14]. The sites of infections may be different but the characteristics (mechanism for biofilm formation) of the causative agent may be similar regardless of the genus. They evade host immune defenses and tolerate treatment with antibiotics. Briefly, formation of biofilm begins with irreversible binding of planktonic bacteria to any surfaces. The bacteria then forms a community which adheres, synthesizes extracellular matrix, matures and disperses around the site. The stages of biofilm development is showed in Figure 1.

The bacteria adheres to the site of infection which has constituents such as electrolytes, water and organic material. The constituents may serve as a source of nutrient for the bacteria. At this stage there will also be planktonic bacteria that is adsorbed to the surface reversibly [16]. At this stage the bacteria is susceptible to antibiotics. The adherence of the bacteria is aided by the pili or flagella and constituents of the bacteria cell i.e. lipopolysaccharide and influenced by the physicochemical properties of the surface. Once the bacteria adhered irreversibly, the bacterial cells colonize the site and divides. At this point, there would be chemical signals being released. These signals suggest formation of a microbial population and there would phenotypic changes to the bacteria. When the bacterial cells senses other surrounding cells in a limited space, there would be secondary signals released (quorum sensing), which leads to autoinduction in the synthesis of extracellular matrix. The biofilm then matures and develop into three-dimensional biofilm structures [17]. The matrix allows the interconnection of immobilized cells which serves as a digestive system and shuts them off from external extracellular enzymes [18,19]. The bottom layers of the biofilm cells are deprived from nutrients and oxygen; hence to have a low metabolic activity [20]. Therefore, these cells are more tolerant to commonly used antimicrobial agents than planktonic cells and treating them becomes difficult [21]. The final stage of the development is detachment. This stage may or may not occur, depending on the environmental condition, nutrients, oxygenation, and other limitations. Cells which detach from the surfaces disperse as liquid or aerosols.

From our previous study [22], we found that silver nanoparticles has bactericidal effect on multidrug resistant *P.aeruginosa*. Therefore, in this study we investigated the effect of silver nanoparticles in the formation of biofilm in multidrug resistant strains of *Pseudomonas aeruginosa.*

## Results and discussion

### Antibiotic profile

Organisms that are resistant to three or more classes of antibiotics are considered as multidrug resistant (MDR). Table 1 shows strains 19, 26, 38, 43a and 47 are MDR strains as they exhibit resistance against carbapenems, cephalosporin, aminoglycosides and fluoroquinolone.

**Table 1:** **Antibiotic susceptibility of *P. aeruginosa *** **strains**

	**Antibiotic class**				
**Strains**	**Carb-apenem**	**Cephalo-sporin**		**Aminogly-coside**	**Fluoro-quinolone**
Antibiotics*	**IPM**	**CFP**	**CAZ**	**GM**	**CIP**
ATCC 27853	29	30	33	21	34
1	29	22	28	21	28
3	**12**	25	28	21	24
6	34	28	33	23	32
11	30	28	30	21	32
19	28	**0**	**0**	**0**	**0**
26	**0**	**10**	**0**	**9**	**0**
38	**0**	**9**	**0**	**8**	**0**
43a	**9**	**0**	**0**	16	**0**
47	21	**12**	**0**	**12**	**0**

*Interpretation according to CLSI; R = resistant; I = intermediate; S = sensitive IPM: R < 14 mm, I:14–15 mm, S >16 mm; CFP: R < 16, I:16–20, S > 20; CAZ: R <15, I : 15–17, S > 17; GM: R <13, I : 13–14,S > 14; CIP: R < 16, I : 16–20, S > 20. Numerics in bold indicate resistant.

### Biofilm formation

**Figure 2:**
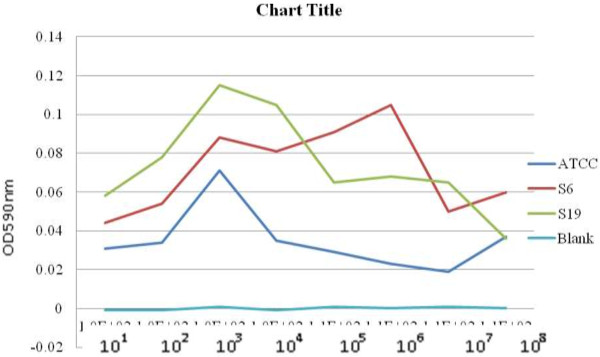
**Biofilm formation in *P. aeruginosa *strains.** Formation of biofilm in three strains of *Pseudomonas aeruginosa*; strain 6 (representative susceptible strain), strain 19 (representative of a multidrug resistant strain) and the ATCC 27853 (American Type Culture collection) strain which was used for comparison. It is observed that the biofilm is formed at bacterial cells of concentration of 10^3^ cfu/ml in both the susceptible and multidrug resistant strain, which is indicated by an elevated value of OD.

Biofilm formation was investigated *in vitro* by monitoring the binding of 0.1% crystal violet to the bacterial cells adhered to a microtiter plate. The optical density value was used as an index to observe the ability to form biofilm in this organism. Figure 2 shows the formation of biofilm in three strains of *Pseudomonas aeruginosa*; strain 6 (representative susceptible strain), strain 19 (representative of a multidrug resistant strain) and the ATCC 27853 (American Type Culture collection) strain which was used for comparison. It is observed that the biofilm is formed at bacterial cells of concentration of 10^3^ cfu/ml in both the susceptible and multidrug resistant strain, which is indicated by an elevated value of OD.

### Inhibition of biofilm formation by silver nanoparticles

**Figure 3:**
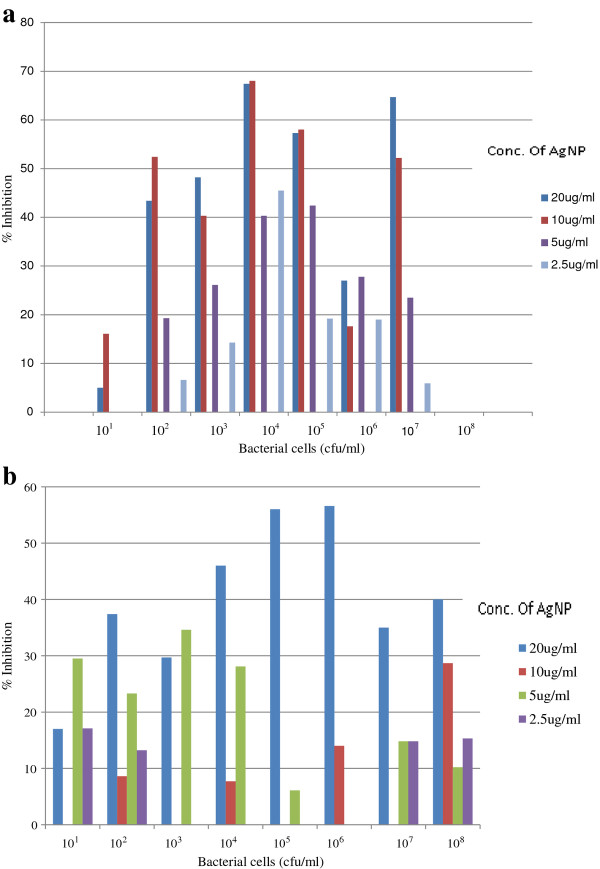
**Activity of silver nanoparticles in *P. aeruginosa *sensitive strain. (a)** Biofilm inhibition assay of sample 6 (representative of susceptible strain). **(b)** Biofilm inhibition assay of sample 19 (representative of multidrug resistant strain).

Figure 3(a) and 3(b) shows the biofilm inhibition assay of sample 6 (representative of susceptible strain) and sample 19 (representative of multidrug resistant strain). The results show that activity of silver nanoparticles is highest at the concentration of 20 μg/ml, with an inhibition rate of 67% and optimal at bacterial concentration of 10^4^ cfu/ml (Figure 3(a)). In the multidrug resistant strain, the inhibition rate of silver nanoparticles was highest at concentration of 20 μg/ml with a bacterial concentration of 10^5^ and 10^6^ cfu/ml. It was also observed that there was no inhibition at bacterial concentration of 10^8^ cfu/ml for all concentration of silver nanoparticles in the sensitive strain (Figure 3(a)). It has been reported that different antimicrobial activities against planktonic bacteria could lead to different extents of biofilm inactivation by the silver nanoparticles. This is evident in Figure 3(a) and 3(b). The penetration rate of the biofilm may also differ between the sensitive and the multidrug resistant strain. The inhibition effect of silver nanoparticles also reduced with the increase in the bacterial cell number. Previous study has also documented that negatively charged silver nanoparticles can be electrostatically repulsed from the negatively charged surfaces of bacterial cells [23]. This suggests that the uptake of the silver nanoparticles could be remarkably reduced as the rate of biofilm formed increased. The ability of silver nanoparticles to agglomerate may also hinder the activity of silver nanoparticles. They may be less efficient in penetrating into the different extent of biofilm.

## Conclusion

This study shows that silver nanoparticles inhibit the formation of biofilm and strains with different antimicrobial activities have also different extent of biofilm formation. It has an effect against multidrug resistant strains of *Pseudomonas aeruginosa.* In order to conclude the mechanism of inhibition of silver nanoparticles in biofilm formation, the mechanism of uptake should be investigated in both the sensitive and multidrug resistant strains.

## Methods

### Characterisation of silver (Ag) nanoparticles

**Figure 4:**
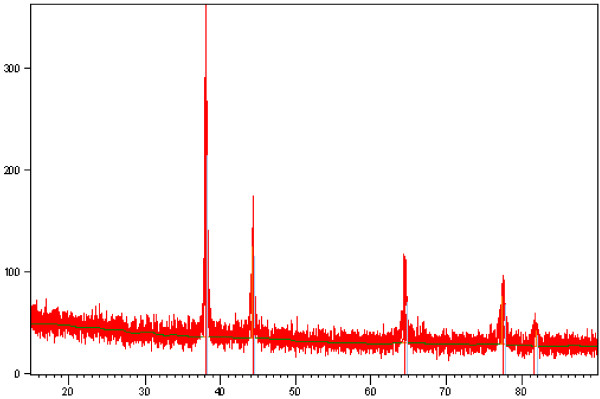
X-ray diffraction pattern for silver (Ag).

Silver (Ag) nanoparticles were purchased from Sigma Aldrich, Sdn Bhd, Malaysia. The nanoparticles used are of sizes that ranged from 20–30 nm. An X-ray diffraction test was carried out on the silver flakes and powder; the phases in Figure 4 show the existence of Ag only, which confirms that the material was pure silver.

### Bacterial strains and culture media

Ten clinical isolates of *P. aeruginosa* strains comprising of five multidrug resistant (MDR) strains were used in the study. Four strains that were susceptible to common empirical antibiotics imipenem (IPM), ceftazidime (CAZ), cefoperazone (CFP), gentamicin (GM) and ciprofloxacin (CIP) *P. aeruginosa* (ATCC 27853) as a control. Brain heart infusion (BHI) and Muller Hinton (MH) agar were used as culture media.

### Antimicrobial susceptibility test

The method used was the agar disk diffusion method as described in Clinical and Laboratory Institute [24]. Approximately 10^8^ cfu/ml of bacterial colonies were lawned onto MH agar plates. Antibiotic discs were placed onto the agar plates. The plates were incubated overnight (18–24 hr) at 37°C. The diameter of zone of inhibition around the disc was observed and recorded. Experiments were conducted in triplicates and average inhibitory zone diameter with its standard deviation was determined.

### Biofilm formation assay

The biofilm assay was carried in a 96-well-flat bottom tissue culture plate (Greiner-bio One, German). Briefly, The bacterial suspension was adjusted to be equivalent to 0.5 McFarland’s standard. A serial dilution was then prepared from 10^8^ cfu/ml till 10 cfu/ml. Each well of the microtiter plate was filled with 150 ul of bacterial suspension. The plate was incubated for 24 h at 37°C. After incubation, the bacterial suspension of each well was gently removed. The wells were washed three times with 0.2 mL of phosphate buffer saline (PBS pH7.2) to remove free-floating ‘planktonic’ bacteria. Adherence of bacteria to the culture plate were stained with crystal violet (0.1%, w/v). Excess stain was rinsed off by washing with deionized water and plates were kept for drying. After drying, 95% ethanol was added to the wells and the optical densities (OD) of stained adherent bacteria were determined with a microplate reader (Spectra-Max 190, USA) at 590 nm. The optical density value was considered as the formation of biofilm on the surface of the culture plate. The experiment was performed in triplicates.

### Biofilm inhibition assay

The bacterial suspension was adjusted equivalent to 0.5 McFarland’s standard. A volume of 100 μl of bacterial suspension was pipetted to each well of a microtiter plate. The silver nanoparticles was diluted two-fold from a stock concentration of 20 μg/ml. The lowest concentration used was 0.06 μg/ml. The diluted silver nanoparticles were then added to the suspension and incubated overnight at 37°C. The method for the quantification of biofilm formation was according to the method of [25]. Briefly, 100 μl suspension of each well was transferred to fresh microtitre plate. The plate was covered, sealed and incubated under stationary conditions at 37°C. After 24 hours incubation, the medium was discarded and thoroughly washed with phosphate buffer saline (pH 7.2). A volume of 100 μl of 0.1% crystal violet was inoculated to each well and left for 30 min at ambient temperature of 25°C. Then, the stain was discarded and the plate was thoroughly washed again. The remaining stain was solubilized with 200 μl of 95% ethanol. A volume of 125 μl of the ethanol solution was then transferred into fresh microtitre plate for analysis at 590 nm.

## Competing interests

All authors have declared no conflict of interest.

## Authors’ contributions

NKP is responsible in the design of the project, coordinationof the project and drafted the manuscript. NF and JH is responsible in the microbiological aspect of the project. ZMZ is responsible in the design and coordination of the project. ANA carried out the molecular studies, and carried out the bacteriological assays. RD is responsible in the design of the project and coordination of the silver nanoparticles preparation. LJP carried out the the optimization of the silver nanoparticles synthesis. All authors read and approved the final manuscript.

## Authors’ information

**Navindra Kumari Palanisamy,** is a senior lecturer and microbiologist affliated to the Faculty of Medicine, Universiti Teknologi MARA, Sungai Buloh, 47000 Sungai Buloh, Selangor, Malaysia.

**Nas Ferina**, is an undergraduate student (Dip in Microbiology, Faculty of Applied Science, UiTM Kuala Pilah), pursuing this project as a fulfilment of her internship.

**Zaini Mohd-Zain** is an Associate Professor and microbiologist affliated to the Institute of Medical Molecular Biotechnology, Faculty of Medicine, Universiti Teknologi MARA, Sungai Buloh, 47000 Sungai Buloh, Selangor, Malaysia.

**Jamal Hussaini** is a senior lecturer and microbiologist affliated to the Faculty of Medicine, Universiti Teknologi MARA, Sungai Buloh, 47000 Sungai Buloh, Selangor, Malaysia.

**Athirah Nur Amirulhusni** is a 3rd Year medical student pursuing her Advance Medical Science (AMS) Programme at Universiti Teknologi MARA (UiTM). She works on antimicrobial activity of silver nanoparticles to gram negative bacteria.

**Rajkumar Durairaj** is Associate Professor at the Head of Department of Mechanical and Material Engineering, Faculty of Engineering and Science, Universiti Tunku Abdul Rahman (UTAR), Malaysia. Prior to joining UTAR, he served as a teaching staff and research assistant at University of Greenwich, UK. He’s research interest is in the area of nanoparticles, rheology and electronic materials. Rajkumar has authored and co-authored a total of 50 technical papers published in peer reviewed journal and leading international conferences. Rajkumar received BEng (1st Class Honours) in Manufacturing Engineering and a PhD from the University of Salford, UK and University of Greenwich, UK, respectively. He is a registered Chartered Engineer with Engineering Council (UK) and Senior Member of the IEEE (US).

**Liew Jian Ping** is a postgraduate student pursuing his Master’s in the area of synthesis and rheological characterisation of silver nanoparticles based dense suspension.
